# Energy-Efficient Single-Stage Nitrite Shunt Denitrification with Saline Sewage through Concise Dissolved Oxygen (DO) Supply: Process Performance and Microbial Communities

**DOI:** 10.3390/microorganisms8060919

**Published:** 2020-06-18

**Authors:** Huichuan Zhuang, Zhuoying Wu, Linji Xu, Shao-Yuan Leu, Po-Heng Lee

**Affiliations:** 1 Department of Civil and Environmental Engineering, Hong Kong Polytechnic University, Hung Hom, Kowloon, Hong Kong, China; zhuanghchit@gmail.com (H.Z.); shao-yuan.leu@polyu.edu.hk (S.-Y.L.); 2 Department of Civil and Environmental Engineering, Imperial College London, South Kensington Campus, London SW7 2AZ, UK; z.wu@imperial.ac.uk; 3 Environmental Engineering Technology Research Center, Chongqing Academy of Ecology and Environmental Sciences, Chongqing 401147, China; dophegood@gmail.com

**Keywords:** nitrite shunt denitrification, 16S rRNA sequencing, dissolve oxygen, total nitrogen removal

## Abstract

Single-stage nitrite shunt denitrification (through nitrite rather than nitrate) with low dissolved oxygen (DO) supply is a better alternative in terms of energy-efficiency, short-footprint, and low C/N-ratio requirement. This study investigates the optimal DO level with temperature effect, with saline sewage at the fixed hydraulic and solids retention times of 8 h and 8 d, respectively. Moreover, 16S rRNA gene sequencing analysis corresponding with total nitrogen (TN) and chemical oxygen demand (COD) removals in each operating condition were performed. Results showed that DO of 0.3 mg/L at 20 °C achieved over 60.7% and over 97.9% of TN and COD removal, respectively, suggesting that such condition achieved effective nitrite-oxidizing bacteria inhibition and efficient denitrification. An unexpected finding was that sulfur-reducing *Haematobacter* and nitrogen-fixing *Geofilum* and *Shinella* were highly abundant with the copredominance of ammonia-oxidizing *Comamonas* and *Nitrosomonas*, nitrite-oxidizing *Limnohabitans*, and denitrifying *Simplicispira*, *Castellaniella,* and *Nitratireductor*. Further, canonical correspondence analysis (CCA) with respect to the operating conditions associated with phenotype prediction via R-based tool Tax4Fun was performed for a preliminary diagnosis of microbial functionality. The effects of DO, temperature, nitrite, and nitrate in various extents toward each predominant microbe were discussed. Collectively, DO is likely pivotal in single-stage nitrite shunt denitrification, as well as microbial communities, for energy-efficient saline sewage treatment.

## 1. Introduction

As the coastal urban population continues to grow at a fast pace, water shortage, energy security, and environmental pollution threats are challenging. Specifically, in coastal cities, seawater intrusion into freshwater aquifers [1] and seawater toilet flush generate high salinity in sewage [2], somewhat causing challenges for its total nitrogen removal. In the traditional biological nitrogen removal (BNR) process, ammonia (NH_4_^+^) is completed oxidized to nitrite (NO_2_^−^) and further into nitrate (NO_3_^−^) in aerobic conditions and then NO_3_^−^ is reduced to nitrogen gas (N_2_) via NO_2_^−^ in anoxic conditions with an electron donor. In biological nitrogen removal from NH_4_^+^ to N_2_, 1 mol NH_4_^+^ requires 2 mol oxygen (O_2_) and 5 mol electron donor in two separate tanks (aerobic and oxic), with an additional recirculation line that is demanding in large-footprint, high energy demand and significant sludge yield.

Alternatively, nitrite-shunt, also known as shortcut nitrification–denitrification, is a better process for saline sewage treatment, as NH_4_^+^ is partially oxidized to NO_2_^−^ and then reduced to N_2_ directly. This requires less oxygen and electron donor demand (1.5 mol O_2_ and 3 mol electron donor per 1 mol NH_4_^+^ to N_2_), saving aeration energy only for partial nitrification, reducing the footprint in a single reactor, and reducing sludge production compared with the traditional nitrification–denitrification process. Complete nitrification is performed in two steps, nitritation and nitratation, accomplished by ammonia-oxidizing bacteria (AOB) and nitrite-oxidizing bacteria (NOB), respectively. The key to nitrite shunt denitrification is to carefully provide a selecting pressure to inhibit NOB for nitration (nitrite to nitrate), while the activity of AOB and denitrifiers should be sustained in one single reactor. Inhibition of NOB, and activation of AOB and denitrifiers, can be mainly controlled by temperature, solids retention time (SRT), and dissolved oxygen (DO) [3]. Raising the temperature of a whole biological system is not energy efficient, although AOB has a higher specific growth rate than that of NOB at higher temperatures [4]. However, SRT and DO together would be a practical solution for limiting the NOB population [5]. AOB could tolerant a lower DO level over NOB, even though the specific growth rate of both decreases at low DO. Meanwhile, denitrifiers should be capable of withstanding DO at a certain low level. As a consequence, at an appropriate combination of low DO level and SRT, AOB and denitrifiers can sustain but NOB cannot, so as to promote denitrification via nitrite shunt. This can be beneficial for energy savings in aeration. Specifically, a low DO supply operation has been reported to increase oxygen transfer efficiency and save up to 16% of energy consumption for aeration, when DO concentration decreased from 2 to 0.5 mg/L [6]. Several nitrite-shunt systems treating high strength wastewaters (e.g., leachate) with low salinity were reported [7,8,9]. For high-saline sewage, the system reported by Capodici et al. achieved 90% chemical oxygen demand (COD) removal and 95% total nitrogen (TN) removal [10]. She et al. reported an oxygen-limited (DO below 1.0 mg/L) nitrification–denitrification sequencing batch reactor treating saline sewage (salinity ranged from 5.0 to 37.7 g NaCl/L), with minimal TN and COD removal of 98.5% and 81%, respectively [11]. The study by Liu et al. shows that NOB was strongly inhibited with 1% salinity of wastewater, while AOB was only moderately inhibited, indicating that partial nitrification is feasible for treating saline sewage [12]. However, most studies focus on high chloride content industrial wastewater. Little information is related to high sulfate content sewage. Nevertheless, at higher saline content operating at a low DO, sulfate can be reduced to hydrogen sulfur by sulfur-reducing bacteria (SRB) through electron donor competition and/or sulfide toxicity towards denitrifiers [13]. Feasible investigation with high saline sewage would be beneficial.

In this study, the feasibility of single-stage nitrite shunt denitrification was conducted with synthetic saline sewage with various DOs (0.5, 0.3, 0.2 mg/L) at a hydraulic retention time (HRT) and SRT of 8 h and 8 d, respectively. The DO levels in the reaction tank were adjusted to 0.5, 0.3, and 0.2 mg/L stepwise. Additionally, the temperature effect (20 and 30 °C) was evaluated. Moreover, the prompt, cost-effective 16S rRNA sequencing data were used to provide a quick understanding of the microbial structure and preliminary microbial functionality so as to map system performance and operations. In this regard, such a demonstration can be used for onsite practitioners to achieve each system optimization.

## 2. Materials and Methods

### 2.1. Reactor Configuration

**Figure 1:**
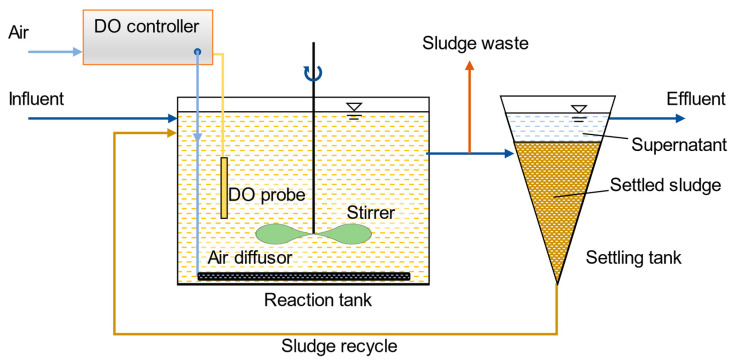
The schematic of the single-stage nitrite shunt denitrification bioreactor.

Figure 1 illustrates a laboratory-scale system consisting of a 2-L reaction tank, a sludge-settling tank, and an auto-DO-controller (Burkert, Ingelfingen, German) for the feasible test of nitrite shunt in total nitrogen removal. The wastewater and sludge were well-mixed by a stirrer in the reaction tank. The auto-DO-controller was regulated by a proportional–integral–derivative calculator (UT350, Yokogawa, Tokyo, Japan), capable of adjusting the air valve responding to the feedback signal from a DO probe (OxySens 120, Hamilton, Reno, NV, USA) for a desired DO level. The water temperature of the reaction tank was controlled by a heating bar (EHEIM, Deizisau, Germany). Settled sludge and effluent was separated in the settling tank. The settled sewage was then returned to the reaction tank, and 250 mL of the mixture in the reaction tank was discharged every day to control the SRT desired (8 d).

### 2.2. Reactor Inoculation, Synthetic Wastewater Composition, and System Operation

The reactor was inoculated with the secondary sludge collected from the sedimentation tank of Shatin Sewage Treatment Works (Hong Kong), with mixed liquor suspended solids (MLSSs) of 2000 mg/L. For simulating the characteristics of Hong Kong’s sewage [14], the influent of the system was the synthetic sewage prepared by mixing 30 mL stock solution (Table S1) with 4.4 L tap water and 1 L seawater (with 2700 mg/L SO_4_^−^) to achieve the desired influent concentrations shown in Table 1. To explore the relationship between system performance and the DO condition, the DO levels in the reaction tank were adjusted to 0.5, 0.3, and 0.2 mg/L stepwise at the temperature of 20 °C, with the fixed SRT and HRT at 8 d and 8 h, respectively. After that, the temperature was increased to 30 °C, while the other conditions stayed the same for investigating the influence of temperature. The duration of each operating period was 40, 15, 23, and 66 days, respectively.

### 2.3. Chemical Analytical Procedures

During the experiment, the effluent samples were collected three times per week. The collected samples were filtered using the 0.45-μm filter before analysis. The COD of the samples was determined by the titration method [15]. The samples were first acidified with 5 mL concentrated sulfuric acid (H_2_SO_4_). Mercuric sulfate (HgSO_4_) was added as a masking agent for chloride ion in the samples. Then, 70 mL of catalyst sulfuric acid-silver sulfate (H_2_SO_4_-Ag_2_SO_4_) and 25.0 mL of 0.25 N of potassium dichromate (K_2_Cr_2_O_7_) solution were added to carry out the 2-h reflux heating program. After heating, the solution was titrated with 0.250 N ferrous ammonium sulfate (Fe(NH_4_)_2_(SO_4_)_2_) using 10 drops of ferroin indicator. Total nitrogen was analyzed using TOC-L analyzers (TOC-LCSH/CPH, Shimadzu, Kyoto, Japan) by 720 °C catalytic thermal decomposition/chemiluminescence methods. NH_4_^+^-N was measured using the indophenol method [16]. The sample was mixed with phenol and sodium hypochlorite alkali solution (alkaline-sodium hypochlorite), using sodium nitrite ferricyanide solution (sodium nitroprusside) as a catalyst to accelerate the coloration. After 30 min in the 35 °C water bath, the reaction generates indophenol. The UV absorption spectra of the solution were determined by spectrophotometer (Libra S35 UV–vis, Biochrom, Cambridge, UK) at a wavelength of 630 nm to obtain the concentration of ammonia. NO_2_^−^ and NO_3_^−^ were measured by an ion chromatograph (DIONEX-100, Thermo Scientific, Waltham, MA, USA) with an IonPac AS9-HC analytical column (Thermo Scientific, Waltham, MA, USA) and a conductivity detector. The waste mixture liquor samples were collected around every two weeks. MLSS and MLVSS of the samples were measured. Then, 50 mL of each waste mixture liquor sample was filtered through a preweighted glass-fiber filter. The solids with filter paper were dried to constant weight using a furnace under 105 °C. Then, the desiccate was heated to 550 °C for 2 h.

### 2.4. Microbial Community Analysis

The microbial community structures of the single-stage nitrite shunt system in the different operating stages were performed based on 16S rRNA gene amplicons sequencing. Genomic DNA was extracted from the sludge samples collected during the experiment (Days 7, 53, 74, 82, and 121) and the inoculum sludge using the PowerSoil DNA Isolation Kit (Mo Bio Laboratories, Carlsbad, CA, USA). The pair of primers 515F (5′-GTGCCAGCMGCCGCGGTAA-3′)/806R (5′-GGACTACHVGGGTWTCTAAT-3′) targeting the hypervariable regions of V4 on bacterial 16S rRNA genes were chosen for polymerase chain reactions of library preparation. The 515F/806R primer set is frequently-used in sludge microbial community analysis according to the study of Wang et al. [17]. 16S rRNA gene amplicons were sequenced using the Illumina HiSeq platform. Table S4 summarizes the amplicon sequencing data statistics. In total, 356,909 high-quality reads of amplicons were generated from all of the samples, with an average length of 253 bp. The paired-end reads obtained from the sequencing platform were merged into contigs, and then low-quality reads were screened using Mothur software (V.1.41.1, Ann Arbor, MI, USA) [18]. The contigs were aligned by the PyNAST method using QIIME (1.9.1) [19,20]. The chimeras in contigs were removed with ChimeraSlayer [21]. In addition, filtered contigs were clustered to the operational taxonomic unit (OTU) at 97% identity, clustering into 12,287 OTUs. Then, taxonomy was assigned to the OTUs with the SILVA rRNA small subunit reference database (SSU123) [22].

### 2.5. Statistics Analysis

The Student’s *t*-test of the reactor performance was conducted using the R function *t*-test for verifying the statistical significance of the results obtained. The genomic functional profiles were predicted by the R-based tool Tax4Fun [23]. Tax4fun predicts the overall genome function based on the abundance of OTU species from flora 16S rRNA gene amplicon sequencing analysis. SILVA (Release 123) was used as the database for 16S marker genes. Tax4fun directly predicts gene function based on the sequence of annotated information through the KEGG prokaryotic genome. Canonical correspondence analysis (CCA) was conducted according to the taxonomy and abundance information of the 16S rRNA gene using the VEGAN package [24]. CCA is the combination of correspondence and multiple regression analysis based on a unimodal distribution model. It is mainly used to reflect the relationship between species and environmental factors. It detects the relationship of environmental factors, samples, and bacterial phases for the important environmental driving factors that affect the distribution of samples.

## 3. Results

### 3.1. Reactor Performance

**Figure 2:**
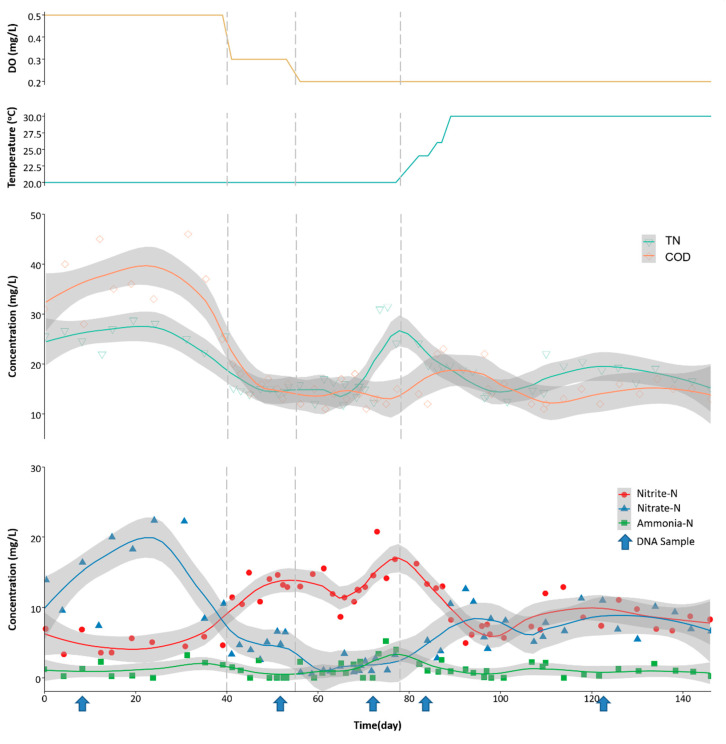
Dissolved oxygen (DO), temperature, and performance of the nitrite shunt system. The yellow line and the green line at the top represent DO and temperature levels. The concentration of nitrogen species and chemical oxygen demand (COD) in the effluent is represented by the scatters. The blue arrows indicate the date of sludge samples collection for 16S rRNA amplicon sequencing. The vertical lines show the separation of each experimental period.

Figure 2 contains the performance of the nitrite shunt denitrification system under various conditions of DO during the whole experiment period. Effluent ammonium was less than 2 mg/L during the whole study, while effluent nitrite increased when DO concentration was 0.3 mg/L (the averages effluent nitrite at DOs of 0.5, 0.3, and 0.2 mg/L were 5.01, 12.83, and 13.61 mg/L, respectively). The average effluent nitrate reduced from 14.93 to 1.60 mg/L when DO dropped from 0.5 to 0.2 mg/L. At the DO of 0.5 mg/L, average effluent COD and TN were about 35.6 and 25.5 mg/L, responding to 86.6% and 31.9% of COD and TN removal efficiencies, respectively. However, at a DO of 0.3 and 0.2 mg/L, the average COD removal increased to 93.9% and 94.8%, respectively, while TN removal improved to 60.7% and 61.1%, respectively. This suggests that low DO (0.3 and 0.2 mg/L) enhanced both COD and TN removal, likely through nitrite shunt denitrification.

After obtaining the optimal DO level (0.3 mg/L) at 20 °C, the temperature of the aeration tank was raised stepwise to 30 °C to simulate sewage temperature transition from winter to spring/summer, while maintaining at the same SRT and HRT of 8 d and 8 h, respectively. As shown in Figure 2, COD removal undulated (effluent COD was around 15 mg/L) in the first few days of the temperature change. However, after the temperature raised to 30 °C, TN removal was slightly higher than that at 20 °C. As the temperature increased, both effluent nitrite and nitrate decreased to around 8 mg N/L, whereas effluent ammonium remained low (<3 mg N/L). This observation indicates that the nitrite shunt system requires a few days for temperature adaption. COD and TN removal efficiencies were similar, whether at 20 or 30 °C under 0.2 mg/L of DO level. Denitrification occurred under both conditions, but higher nitrate in the effluent was observed at 30 °C than at 20 °C. Moreover, the high abundance of denitrifiers was probably responsible for COD removal due to the organic matter serving as electron donors for denitrification via nitrite or nitrate. Worthy of note is that DO of 0.3 mg/L did not negatively affect nitrite shunt, suggesting that such low DO dosage may not affect nitrite shunt denitrifiers. Adversely, when the DO concentration decreased from 0.3 to 0.2 mg/L, TN removal reduced. This may be caused by insufficient oxygen for effective ammonia oxidation to nitrite.

The statistical significance of the experimental data was verified by means of a Student’s *t*-test (Table S3). Among different DO levels, NO_2_^−^-N, NO_3_^−^-N, TN, and COD showed a significant difference in the comparison of 0.5 versus 0.3 mg/L and 0.5 versus 0.2 mg/L, while NO_3_^−^-N, NH_4_^+^-N, and COD showed a significant difference in the comparison of 0.3 versus 0.2 mg/L. As to a temperature comparison, a significant difference was observed in NO_2_^−^-N and NO_3_^−^-N.

Table 2 summarizes the removal rates of TN, NH_4_^+^, and COD of the system. The average concentrations of nitrogen compound, COD, MLSS, and MLVSS are summarized in Table S2. The highest TN removal (approximate 60%) was achieved with ammonia removal of 97.9% at 0.3 mg/L DO and 20 °C. The results of TN and nitrogen composition displayed nitrite accumulation when the DO was lower than 0.3 mg/L, indicating that NOB was inhibited under such DO environments. This shows that nitrite shunt can be achieved at a low DO condition (>0.3 mg/L) with saline sewage.

### 3.2. Microbial Community Changes Influenced by Operation Conditions

To reveal the evolution of the microbial community during the experiment, five sludge samples on different periods (Days 7, 53, 74, 82, and 121) and one inoculum sludge were collected for 16S rRNA genes amplicon sequencing. All the reads were classified into 52 phyla and 1712 genera, respectively. At the phylum level (Figure S1), the most abundant phyla in the system were *Proteobacteria* (66.90% on average), *Bacteroidetes* (24.87% on average), and *Firmicutes* (2.38% on average). These three phyla counted for 94.15% of the total bacteria in all of the samples. The relative abundance of *Proteobacteria* in the system was decreased from 73.4% (Day 7) to 58.21% (Day 121), while the relative abundance of *Bacteroidetes* increased from 20.18% to 31.63% and that of *Firmicutes* increased from 1.09% to 6.84% in the same period.

**Figure 3:**
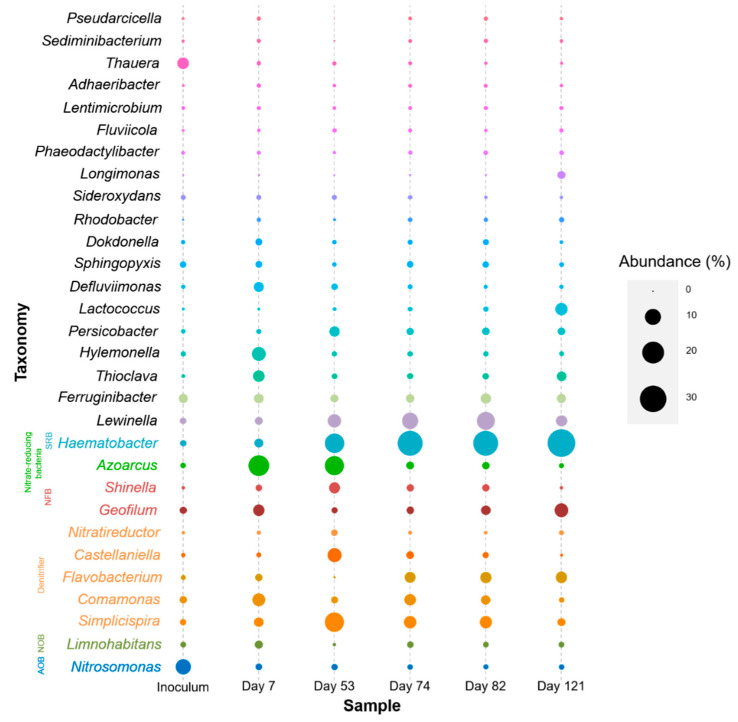
Microbial communities at various conditions at the genus level. The size of bubbles represents the abundance of genera. The colors of generic names represent their functional groups.

Figure 3 illustrates the variations of microbial communities at the genus level with the different operating conditions. At the beginning of the reactor operation (Day 7), the most predominant genus in the system was facultatively anaerobic nitrate-reducing *Azoarcus* (17.91%) that uses nitrate as the electron acceptor [25]. However, its abundance decreased to only 0.75% on Day 121. Adversely, while *Azoarcus* declined in the system, anaerobic sulfate-reducing *Haematobacter* for sulfide formation [26] became the most predominant genus. Its relative abundance increased from 2.88% on Day 7 to 32.47% on Day 121. Furthermore, denitrifying *Simplicispira*, *Castellaniella*, and *Nitratireductor* were also identified [27,28,29]. The abundance of these denitrifiers peaked on Day 53 under the DO of 0.3 mg/L (14.97%, 7.72%, and 1.41%, respectively). Nevertheless, the other two denitrifiers, *Flavobacterium* and *Comamonas* [30], were low in abundance on Day 53 (0.07%) but increased afterward. *Nitrosomonas* was identified as AOB [31]. The information of *Limnohabitans* sp. 103DPR2 from the KEGG database shows that this genus has the potential function of autotrophic nitrite oxidation [32]. Although it does not belong to one of the seven known genera that are able to perform nitrite oxidation [33], *Limnohabitans* was classified as NOB in this study. As expected, with the DO level decreased, the abundance of both AOB and NOB showed the trends of decreasing. From Day 7 to 121, the abundance of *Comamonas*, *Nitrosomonas*, and *Limnohabitans* decreased from 6.20%, 1.46%, and 2.17% to 0.89%, 0.87%, and 1.17%, respectively. Chemo-organotrophic *Lewinella* and *Ferruginibacter* increased gradually during system operation [34,35]. Unexpectedly, some genera with the ability of nitrogen-fixation, such as *Geofilum* and *Shinella* [36,37], were identified.

**Figure 4:**
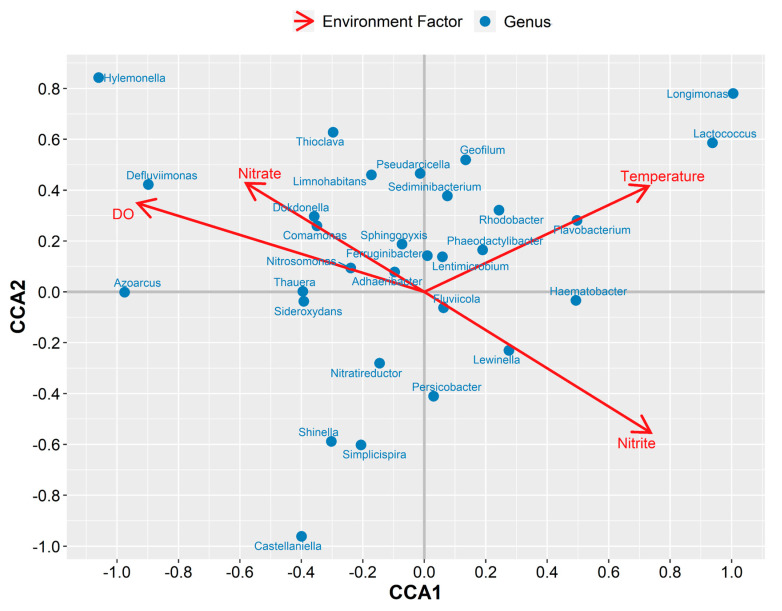
Canonical correspondence analysis (CCA) of the microbial community with respect to the operating conditions. CCA1 and CCA2 represent the first two ordination axes. The blue dots represent the top thirty genera in abundance, and the red arrows represent environment factors in the systems.

To better understand the correspondent association between microbial communities and the operating parameters, i.e., DO level, temperature, and effluent nitrate/nitrite, canonical correspondence analysis (CCA) was performed (Figure 4). The eigenvalues of the first two CCA axes (CCA1 and CCA2) were 0.2629 and 0.1478, respectively. In the system, a few microbes (*Hylemonella, Defluviimonas, Dokdonella, Comamonas, Nitrosomonas*, and *Adhaeribacte*) were positively correlated with DO level and effluent nitrate. Among those genera, the *Comamonas* and *Nitrosomonas* are AOB, whereas *Simplicispira*, *Castellaniella*, and *Nitratireductor* are denitrifiers. *Lewinella* and *Fluviicola* presented some relationship to effluent nitrite. Jointly, these results identified that DO plays the most vital role in nitrite shunt denitrification with the synthetic saline sewage.

### 3.3. Phenotype Prediction

**Figure 5:**
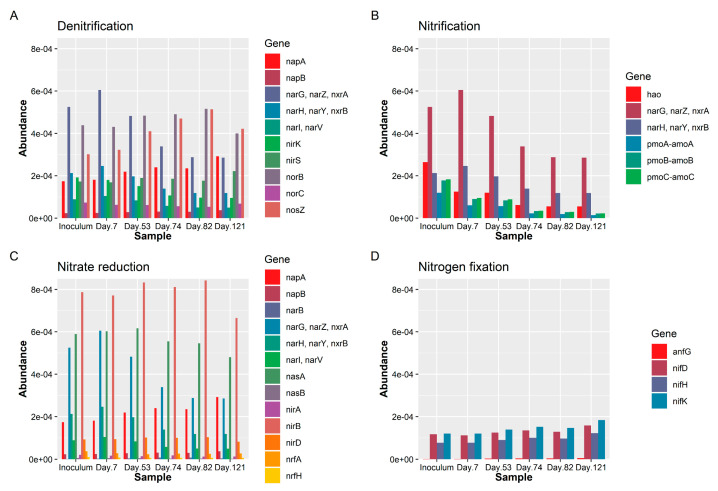
Gene abundance for nitrogen metabolism. (**A**) The abundance of nitrification-related genes. (**B**) The abundance of denitrification-related genes. (**C**) Abundance of nitrification-related genes. (**D**) The abundance of nitrogen-fixation-related genes.

Although the abundance variations in the microbial community (genotype) somewhat provide clues in the system, their phenotypes were missed among over 1700 genera. To this end, the R-based Tax4Fun tool, which somewhat offers, even if imprecisely, the potential functions and metabolisms based on 16S rRNA gene amplicon sequencing datasets, was used. Figure 5 (Figure 5A–D) shows the variation of predicted genes related to nitrogen transformation for the nitrite shunt denitrification system. The abundance of genes encoding nitrification-related enzymes, including hydroxylamine oxidoreductase (*hao*), ammonia monooxygenase (*amoABC*), and nitrite oxidoreductase (*nxrAB*), declined when DO level decreased from 0.5 to 0.3 mg/L [33,38,39]. However, changes were negligible between the samples collected on Day 82 (0.2 mg/L DO; 20 °C) and Day 121 (0.2 mg/L DO; 30 °C), indicating that nitrification may not be influenced by the operation temperature (20 and 30 °C). The most abundant genes for denitrification, except nitrate reductase (*narGHI*), increased when DO decreased from 0.5 to 0.3 mg/L (Figure 5B) [40]. The genes NADH-dependent nitrite reductase (*nirDB*) and respiratory nitrite reductase (*nrfAH*), related to nitrite reduction to ammonia, did not change much among the operating parameters tested [41]. Notedly, some abundance of nitrogen fixation associated genes, such as Fe-only nitrogenases (*anfG*) and Mo-nitrogenases (*nifDHK*), slightly increased during the whole experiment.

## 4. Discussion

In this study, the optimal TN removal efficiency (60.67%) was achieved under the condition of 0.3 mg/L DO, 20 °C, 8 d SRT, and 8 h HRT. DO (0.3 mg/L) shows an effective strategy for coupling NOB inhibition and AOB and denitrifier activation. Under such a condition, although the abundance of AOB, NOB, and denitrifiers decreased, the activity of ammonia oxidization and denitrification (but not nitrite oxidation) in the system was maintained at a high level, compared to the highest and lowest DO level tested (0.5 and 0.2 mg/L, respectively). Compared to the studies of She et al. [11], the system in this study had similar COD and ammonia removal. However, the TN removal in this study (~60%) is lower than their system (98.5%). It is worth noting that the influent of She et al.’s system contained little sulfate.

This raises a question: why was the TN removal rate not able to improve further? Under the condition of 0.3 mg/L DO and 20 °C, nitrite accumulation (12–13 NO_2_^−^-N mg/L and 2 NH_4_^+^-N) is likely responsible for such a limit. One hypothesis is that COD was inefficient for further denitrification. Indeed, we observed the relatively high abundance of sulfate-reducing *Haematobacter* (over 30%), which supports its high carbon organics affinity over the nitrite shunt denitrifying bacteria (around 8%) under the coupling effect of DO (0.2 mg/L) and sulfate levels (160–180 mg/L). The domination of *Haematobacter* corresponding to this interspecies competition under such low DO levels is somewhat different from the observation by Ryoko et al., which reported that denitrifiers are dominant under anoxic conditions, but sulfate reducers are outcompeted under anaerobic conditions [42]. Another hypothesis is that nitrogen fixation occurred. Certainly, the nitrogen-fixation-related genes (*anfG* and *nifDHK*) predicted by the Tax4Fun analysis increased, suggesting that they could probably convert dinitrogen gas into ionic nitrogen so that total nitrogen flux increased in the system. If so, a proportion of carbon organics in influent were taken up by nitrogen fixation rather than nitrite shunt denitrification, which led to the limited TN removal. Furthermore, a few of the nitrogen-fixation bacteria metabolize photosynthetically [43], as was observed via 16S rRNA gene sequencing, where the photosynthesizing-bacteria *Rhodobacter, Azoarcus, Geofilum*, and *Shinella* [36,44,45,46] were shown to coexist in this system. This was probably due to the reactor not being deliberately protected from light. Collectively, these findings deserve further investigation.

A considerable microbial database of ecological and engineered environments that could offset the lack of functional genes information in the 16S rRNA genes systems has been accumulated over the years. Moreover, 16S amplicons sequencing analysis followed by the R-based Tax4Fun tool should serve as a preliminary diagnosis of microbial metabolism and interactions [47], albeit from an approximate perspective, leading to the consideration of whether further metagenomics and/or metatranscriptomics techniques are necessary. In any event, this demonstration offers lower taxonomic ranks and raises overlooked genetic potentials for screening as to whether metagenomics and metatranscriptomics are necessary to further disclose indepth information.

The MLSS and MLVSS values obtained (Table S2) have a non-negligible standard deviation due to the inadequate sampling frequency. Please note that the MLSS and MLVSS thus provide only a rough index and not an accurate reflection on the growth of sludge. Moreover, the periods of the second and third stages (15 and 23 days, respectively) were not sufficient for the pseudo-steady state. However, this may represent short fluctuations in the operation of real systems and reflect the responses of the microbial community.

## 5. Conclusions

Nitrite shunt denitrification with saline sewage was investigated in different DOs (0.5, 0.3, and 0.2 mg/L) and temperatures (20 and 30 °C). TN and COD removals, along with their respective microbial communities (16S rRNA sequencing), were studied. Reactor performance indicates that DO control (0.3 mg/L) could be an effective strategy for nitrite shunt denitrification with saline sewage. Under the condition of DO of 0.3 mg/L, 60.7% TN removal and 97.9% COD removal were achieved. Although the data in this study could not accurately represent pseudo-steady state conditions (due to the short time periods of the second and third stages), such short operating periods could be similar to short fluctuations and reflect the response of microorganisms in real systems. The latter should be taken into consideration during the interpretation of the results, especially concerning overall system performance. The abundance of denitrifiers increased when the DO level decreased in the system. However, the activity of AOB was sufficient to convert the ammonia in the influent to nitrite. As a consequence, the TN and COD removal increased, and nitrite accumulation was observed in the system. This study not only displays the feasibility of nitrite shunt denitrification with saline sewage but also discloses its microbial structure and interactions.

## Figures and Tables

**Table 1:** The synthetic wastewater composition.

Item	Concentration
Chemical oxygen demand (COD)	265 mg/L
Ammonium	30 mg N/L
Total nitrogen (TN)	38–39 mg N/L
Seawater	18%
Cl^−^	3700–3900 mg/L
SO_4_^2−^	160–180 mg/L

**Table 2:** Summary of reactor performance as a function of DO and temperature.

Time (day)	DO Level (mg/L)	Temp. (°C)	TN Removal (%)	NH_4_^+^ Removal (%)	COD Removal (%)
0–40	0.5	20	31.89 ± 5.63	95.73 ± 3.42	86.57 ± 2.44
41–55	0.3	20	60.67 ± 1.36	97.91 ± 2.96	93.92 ± 0.79
56–78	0.2	20	52.92 ± 16.67	94.09 ± 5.15	94.80 ± 0.76
79–146	0.2	30	54.52 ± 7.24	97.01 ± 2.35	91.72 ± 6.60

## Supplementary Materials

The following are available online at https://www.mdpi.com/2076-2607/8/6/919/s1, Table S1: The synthetic stock solution; Table S2: Summary of reactor performance on average; Table S3: Results of statistical comparison by means of a Student’s *t*-test; Table S4: Data statistics of the 16S rRNA gene amplicon sequencing; Figure S1: Microbial communities at various conditions at the phylum level. The sequencing data have been deposited in the NCBI short reads archive database (accession number: PRJNA606970).
