# Supplementary material for: Energy-Efficient Single-Stage Nitrite Shunt Denitrification with Saline Sewage through Concise Dissolved Oxygen (DO) Supply: Process Performance and Microbial Communities
Source: Microorganisms. 2020 Jun 18;8(6):919. doi: 10.3390/microorganisms8060919 (PMC7356877; doi:10.3390/microorganisms8060919)
Supplement: Supplementary file 1 [file microorganisms-08-00919-s001.pdf]

Energy-efficient single-stage nitrite shunt denitrification with saline sewage through concise dissolved oxygen (DO) supply: Process performance and microbial communities

Huichuan Zhuang [1], Zhuoying Wu [2], Linji Xu [3], Shao-Yuan Leu [1], Po-Heng Lee [1,2,*]

1. Department of Civil and Environmental Engineering, Hong Kong Polytechnic University, Hung Hom, Kowloon, Hong Kong, China
2. Department of Civil and Environmental Engineering, Imperial College London, South Kensington Campus, London, UK
3. Environmental Engineering Technology Research Center, Chongqing Academy of Ecology and Environmental Sciences, Chongqing, China

Table S1. Synthetic stock solution

Substrate	Concentration
Glucose	19.57 g/L
Sodium acetate	26.1 g/L
Yeast extract	9.786 g/L
NH_4Cl	18.37 g/L
K_2HPO_4	1.92 g/L
KH_2PO_4	0.72 g/L
$MgCl_2$ $6H_2O$	8.32 g/L
$CaCl_2$	5.2 g/L
Trace solution	2.50 mL/L

Table S2. Summary of reactor performance in average.

Time (day)	DO level (mg/L)	Temp. (°C)	MLSS (mg/L)	MLVSS (mg/L)	N-NO_2^- (mg N/L)	N-NO_3^- (mg N/L)	N-NH_4^+ (mg N/L)	TN (mg/L)	COD (mg/L)
0-40	0.5	20	2465 ±409	2025 ±335	5.0±1.3	14.9±5.7	1.3±1.1	25.5±2.2	35.6±6.8
41-55	0.3	20	2293 ±346	1827 ±120	12.8±1.7	4.7±1.4	0.6±1.0	14.8±0.6	16.1±2.2
56-78	0.2	20	2193 ±175	1893 ±98	13.6±2.9	1.6±1.0	1.8±1.6	17.7±6.5	13.8±2.1
79-146	0.2	30	2385 ±409	1930 ±395	8.1±2.1	8.1±2.4	0.9±0.7	17.1±2.8	22.0±18.0

Table S3. Results of statistical comparison by means of a Student's t-test

	Comparison DO = 0.5 VS. DO = 0.3 (20°C)	Comparison DO = 0.3 VS. DO = 0.2 (20°C)	Comparison DO = 0.5 VS. DO = 0.2 (20°C)	Comparison 20°C VS. 30°C (DO = 0.2)
Nitrite-N	t = -10.562<br>p = 1.07×10^{-7}	t = -0.78945<br>p = 4.40×10^{-1}	t = -9.7253<br>p = 7.41×10^{-9}	t = 6.0414<br>p = 3.94×10^{-6}
Nitrate-N	t = 5.4557<br>p = 2.48×10^{-4}	t = 5.4891<br>p = 1.95×10^{-4}	t = 7.287<br>p = 3.74×10^{-5}	t = -10.517<br>p = 1.15×10^{-10}
Ammonia-N	t = 1.3643<br>p = 1.92×10^{-1}	t = -2.0998<br>p = 4.87×10^{-2}	t = -0.89226<br>p = 3.82×10^{-1}	t = 1.8988<br>p = 7.48×10^{-2}
TN	t = 14.787<br>p = 2.76×10^{-8}	t = -1.6671<br>p = 1.19×10^{-1}	t = 4.2148<br>p = 5.85×10^{-4}	t = 0.32396<br>p = 7.50×10^{-1}
COD	t = 8.5<br>p = 3.00×10^{-6}	t = 2.4226<br>p = 2.97×10^{-2}	t = 9.8184<br>p = 1.60×10^{-6}	t = -1.9619<br>p = 6.49×10^{-2}

In Student's t-test, t = t-value, p = p-value (statistically significant difference if $p < 0.05$)

Table S4. Data statistics of the 16S rRNA gene amplicon sequencing

Sample name	Total reads	Minimum length (bp)	Maximum length (bp)	Average length (bp)	classified reads	assigned sequences
Inoculum	54,736	240	377	253	54,716	54,691
Day 7	55,244	239	369	253	55,215	55,171
Day 53	57,641	240	340	253	57,546	57,524
Day 74	69,810	158	390	253	69,783	69,729
Day 82	63,987	226	388	253	63,928	63,878
Day 121	55,491	152	390	253	55,476	55,425

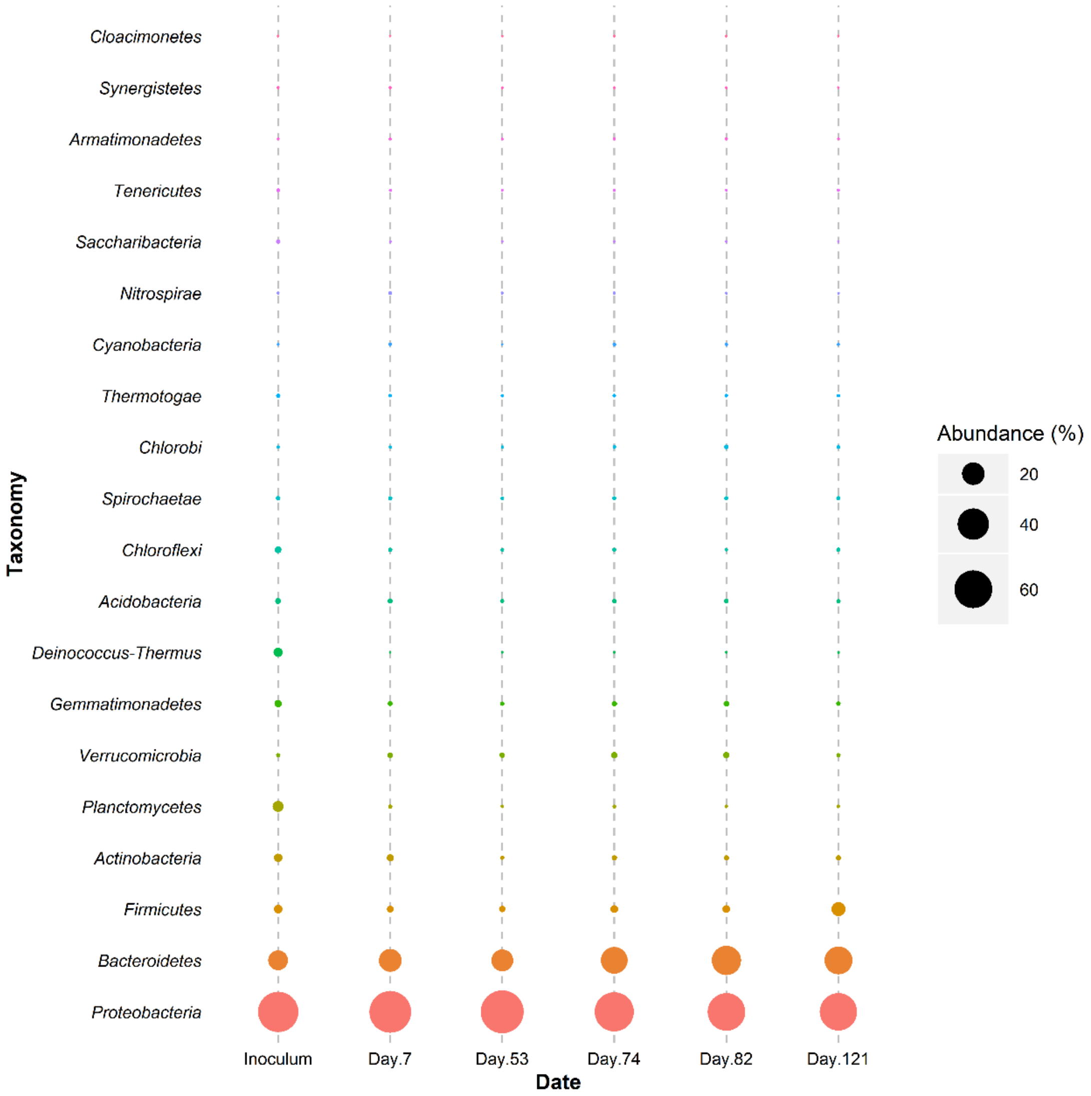

Figure S1 Microbial communities at various conditions at phylum level
